# EURL ECVAM Literature Review Series on Advanced Non-Animal Models for Respiratory Diseases, Breast Cancer and Neurodegenerative Disorders

**DOI:** 10.3390/ani12172180

**Published:** 2022-08-25

**Authors:** Laura Gribaldo, Adelaide Dura

**Affiliations:** European Commission, Joint Research Centre (JRC), 21027 Ispra, Italy

**Keywords:** in vitro models, biomedical sciences, human-based models

## Abstract

**Simple Summary:**

Directive 2010/63/EU on the protection of animals used for scientific purposes sets out the legal requirements for implementing the ‘Three Rs’ principles of Replacement, Reduction and Refinement of animal procedures. The final goal is to phase out animal testing and replace it with scientifically valid non-animal alternatives. According to the latest statistics, in 2019, the European Union used approximately 10 million animals in experimental procedures with about 70% of those used for biomedical research. However, effective new therapies for several serious diseases are still lacking. Over 90% of new drugs fail to progress to the market due mainly to a lack of efficacy or unexplained toxicity. This suggests that reliance on animal models is failing to identify novel therapies. In this context, the EU Reference Laboratory for alternatives to animal testing (EURLECVAM) of the European Commission’s Joint Research Centre carried out a series of studies to produce a unique knowledge base that describes in detail non-animal models applied in several biomedical research areas. Here, a summary of the results on the areas of respiratory tract diseases, breast cancer and neurodegenerative disorders is described and commented on.

**Abstract:**

In vivo models are used in biomedical research to reproduce human disease and develop new drugs. However, they do not mimic the disease as it occurs in humans, and their use has failed to identify novel therapies effective for many highly prevalent non-communicable diseases, such as Alzheimer’s disease. Indeed, the clinical failure rate in drug development remains very high, with an overall likelihood of approval from Phase I of about 9.6%. On the other hand, human-based models, advanced imaging techniques and human epidemiological studies may increase our understanding of disease aetiology and pathogenesis and enable the advance of safe and effective therapies. Particularly when human tissues are used, they may produce faster, cheaper results, more predictive for humans, whilst yielding greater comprehensions of human biochemical processes. A first effort to collect existing knowledge about non-animal models of highly prevalent human diseases was made by the Joint Research Centre of the European Commission. The final aim was to identify and share information on the capabilities and limits of human-based models at different levels: scientific communities, universities and secondary schools, national committees for animal welfare and the public at large.

## 1. Introduction

The use of animal models to advance science and medicine has been a quarrelsome subject for many years. Although they contributed to several scientific achievements, the concerns about such studies on both ethical and scientific grounds have found strength with the increase in new advanced approaches, which do not use animals. In the European Union (EU), and perhaps elsewhere, basic and applied research accounts for most of the animals used in science, as indicated in the most recent statistical report on animal use in EU Member States [1]. Nevertheless, these models may yield results that cannot always be translated into the human in vivo situation. The physiology of rodents, the animal species most commonly used in research, differs significantly from that of humans in many respects. Consequently, around 90% of drugs fail in clinical trials, particularly in the area of cancer research [2] and Alzheimer’s disease [3]. This is mainly due to unexpected toxicity or lack of efficacy in patients that were not picked up by preclinical animal tests. Therefore, scientific concerns about the predictive power of animal models are driving the development of animal-free, human-relevant approaches in different branches of life sciences.

In this light, the European Union Reference Laboratory for alternatives to animal testing (EURL ECVAM) of the European Commission’s Joint Research Centre carried out a series of studies on available and emerging non-animal models in seven areas: respiratory tract diseases, neurodegenerative disorders, breast cancer, immune-oncology, autoimmunity, cardiovascular diseases and immunogenicity of advanced medicinal products. The areas were selected based on consideration of disease incidence and prevalence and the number of animal procedures conducted [4].

The result is a unique, highly curated knowledge base containing detailed descriptions of non-animal models being used in each of the disease areas, including information on their applications and biological relevance. Researchers would use the knowledge base to search for models that can be refined and used to tackle their own research questions. Educators could inform their students about the latest information on the state of the art currently available, while funding bodies can identify trends and target areas for investment. Furthermore, the knowledge base will be of use to Competent Authorities, as referred to in Directive 2010/63/EU for the protection of animals used for scientific purposes, to support the process of project evaluation.

Below is a summary of the meta-analysis previously conducted on the information collected and published in three areas of interest: respiratory diseases, breast cancer and neurodegenerative disorders.

## 2. Methodology

A customised strategy was developed for each study to obtain a collection of relevant non-animal models [5,6,7]. The strategy was based on agreed inclusion and exclusion criteria and used a predefined set of search terms. In addition, grey literature, such as news, events, societies, etc., was also considered.

## 3. Respiratory Tract Diseases

Lung illnesses such as asthma, chronic obstructive pulmonary disease (COPD), chronic respiratory infections, pulmonary fibrosis and lung cancer are major existing health problems. Current models of respiratory disease rely greatly on in vivo endpoints including (histo)pathology, histology and mediator analysis and, in diseases such as asthma, measures of airway (hyper)responsiveness. The development of animal models of respiratory disease often bears little resemblance to human pathology. For example, mice do not develop asthma naturally, and therefore, in order to generate an animal model with symptoms of the disease, a protein such as ovalbumin is instilled into the airways to induce the condition artificially [8]. Whilst some animal models may recapitulate some of the features of the disease, the absence of active new therapies for severe respiratory disorders, such as asthma, indicates that reliance on animal models is failing to identify disease mechanisms that could lead to novel therapies. Within the biomedical research domain, there is currently little international standardisation, with most research bodies and laboratories establishing their own methods for conducting novel research across a wide array of disciplines. The EURL ECVAM study [5] was based on a comprehensive review of peer-reviewed scientific literature. Over 21,000 abstracts (11,636 non-cancer and 9421 cancer) were scanned for relevant non-animal models of respiratory disease. From this, 284 publications were identified as promising candidate methods according to defined criteria. The review found that historically, the predominant non-animal models used for respiratory disease research have been based on simple in vitro cell cultures. Such models are still prominent and have their uses, particularly because they are inexpensive and easy to work with. However, in the past five years or so, research focus has been shifting towards increasingly sophisticated bioengineering approaches that recapitulate lung development, form and physiologic functions in vitro. Many of these are based on 3D cultures, spheroids, organoids and microfluidic or ‘lung-on-chip’ systems. Several publications describe general models and methods in the initial phases of development, with the aim of demonstrating reproducibility and prospective application in many disease areas. These include sources of human epithelial cells; lung models based on human pluripotent stem cells; novel microfluidic devices using organoid models that mimic the lung microenvironment during homeostasis and disease states; and methods for studying respiratory absorption. Of the 51 biological biomarkers or ‘endpoints’ identified in the review, those based on the detection of key proteins or measurements of gene expression are the most commonly used across all methods. Cell viability, migration and metabolism are key endpoints used for in vitro lung cancer studies. Finally, a series of recommendations have been identified, e.g., encouraging the development of Standard Operating Procedures (SOP) for model generation and testing as well as stimulating a more productive dialogue between the fields of oncology and respiratory diseases. 

## 4. Breast Cancer

Breast cancer is the most common cancer among women in the European Union and worldwide [9]. It differs significantly among patients and even within every single tumor. Despite advances in early detection, approximately 30% of all patients have recurrent disease, which is metastatic in most cases [10]. Preclinical breast cancer research currently relies on animal models, mostly rodents. However, animal models mimic limited aspects of human breast cancer. To offer better treatment with improved efficacy, it is necessary to select therapies based on the patient and the clinical and molecular features of the tumor; therefore, human-based models can better address the heterogeneity of human breast cancer. The EURL ECVAM study [6] comprised an extensive review of the scientific literature published from January 2014 to March 2019 that resulted in 935 identified papers describing relevant non-animal models for breast cancer. In vitro models based on a variety of immortalised cell lines resulted to be the most representative approach used for breast cancer. These cell lines are frequently commercially accessible or already qualified lines. While 2D cell lines are convenient research models to study breast cancer, they are relatively simplistic; therefore, the use of 3D culture conditions has increased over time reaching in 2018 the publication level of 2D models. A 3D dimension can better mimic the complexity and heterogeneity that characterise human breast. The use of a scaffolding system appeared as the leading approach employed in 3D models, followed by organoids and spheroids, such as mammospheres. Different culture conditions were also observed in combination with microfluidic systems. It was also observed that in silico methods are often used in combination with in vitro models. This still represents a minor niche, but it is worth highlighting their applications to most of the breast cancer disease features. The use of machine learning in predictive modelling is a very interesting approach, especially in drug development and for testing new therapeutic strategies. Although 5% to 10% of breast cancer could be hereditary, the high incidence highlights the lack of knowledge about the events triggering breast cancer initiation and whether it skips immunological recognition. This review found that non-animal models are employed mainly to study cancer initiation and development in order to fill this gap and discover the molecular bases of the driving events. 

Seeing the whole outcome of the review, the key assumptions were that the use of non-animal models in human breast cancer research is widely extended, especially for cell-based in vitro models. They are generally applied to investigate disease mechanisms and to assess drug candidates. In particular, human-based models focused on studying breast cancer initiation and development and pharmacological and/or physical treatments. Qualified and commercially accessible human breast cancer immortalised cell lines represent the most used models. Nevertheless, immortalised cells are frequently cultured in 3D conditions, such as spheroids, to better mimic breast cancer pathophysiology. 

## 5. Neurodegenerative Diseases

Neurodegenerative diseases are incurable and debilitating conditions that result in progressive degeneration or death of nerve cells. They consist of Alzheimer’s disease and other dementias, Parkinson’s disease (PD), Huntington’s disease, motor neuron disease, Creutzfeldt–Jakob disease and multiple sclerosis. Of these, dementias account for the greatest burden of disease [11], with Alzheimer’s disease (AD) representing over 60–70% of the cases. There is currently no cure for AD [12], but some symptomatic treatments are available. Animal disease models are considered important in the development of new drugs, but, unfortunately, none of the current animal models of Alzheimer’s disease has either construct or predictive validity. Ultimately, the hypothesis can only be tested in human patients and only with the proper tools such as pharmacologically active intervention and clinical trials suited to evaluate the mechanism of action. Integration of knowledge in quantitative models is considered also important, if not essential, in this process. The EURL ECVAM collection describes 568 different models, compiled from 519 publications published between 2013 and 2018 [7]. Models based on primary cells or stem cells represent the highest fraction for PD, whereas biochemical/cell-free models represent the most commonly used group of methods for AD, followed by human/patient ex vivo models (22%) and computational models (18%). Ex vivo human/patient material was primarily used to evaluate aspects of protein aggregation, in the case of PD and amyloid β and tau accumulation in the case of AD. Despite this strong focus on protein deposits, disease diagnosis was not the main aim of most of these methods/models but rather developing a better understanding of the disease mechanism. Unsurprisingly, the overall novelty, throughput and content of these methods were rather limited, as ex vivo material by definition has limited accessibility. While human/patient primary or stem-cell models were clearly the subject of intense research, this field is still under full development. The largest part of the inventoried methods consists of induced pluripotent stem cells (iPSCs). The most striking difference between the AD and PD fields is the relative shift in application. Where the majority of primary or stem-cell models are used for mechanistic studies in AD, there is a larger focus on treatment and therapy (dopaminergic cell replacement) when it comes to PD. Human-derived cell lines are less specific models, especially when compared to the aforementioned two categories (ex vivo material and primary or stem cells). However, they still represent an important part of the models and methods currently used in these fields.

At the same time, throughput and content (i.e., biological representation) are rather low. The most known or common examples of 2D or 3D co-culture models are the ones representing blood–brain barrier function, but a standard method does not appear to be available yet. When it comes to the disease feature addressed, the majority of co-culture or 3D models are still of exploratory nature. There is a need for physiological, multifunctional and architectural complex systems representing the central nervous system (CNS) or brain tissue, though improved characterisation of model systems is required in advance to mechanistic studies on disease features. Organoids in general are highly relevant since they may enable cell-based human models capable of supplementing the need for animal-based models. The inventoried methods are, however, very limited thus far. One area of organoid development currently receiving attention is the need for vascularisation of organoids. In order to model increasingly complex brain-like tissue structures, this will need to be addressed. 

Biochemical and cell-free model systems comprised 65 models in the AD area, 16 models in the PD area and 17 models in both PD and AD, and/or in general for neurodegenerative disease (NDD). One of the most prominent observations for this type of method/assay is their clear focus on protein aggregation. This should not be surprising, seeing the high relevance of protein misfolding in the pathology of many different neurodegenerative diseases and the suitability of biochemical and cell-free systems to study (aspects of) the physiological and pathological processes leading up to protein misfolding and aggregation. A striking difference across the two disease areas included is the status of the methods, with the AD field showing markedly more developmental efforts in this regard, compared to PD research. Lab/brain-on-chip or microfluidic systems comprised 40 models in the AD area, two models in the PD area and nine models in the NDD area. Many of the AD-focused models were aimed at disease diagnosis (approx. 44%). There was a wide range of detection limits (spanning fg/ml to hundreds of ng/mL) and poor definition of the biofluids in which amyloid β was isolated and quantified. Future developments may include bringing such diagnostic devices into the clinical setting and defining minimum acceptable limits of detection. With regard to the disease areas of several NDDs and PD, again, a majority of the devices were dedicated to the study or quantification of protein aggregation. Across all disease areas, microfluidic models tended to exhibit high throughput but medium-to-low content. The ability of these models to achieve high throughput may be one reason the model type is valuable in the context of augmenting or supplanting animal studies. Finally, in silico models are highly focused on the investigation of disease mechanisms or as disease models, with more than two-thirds of the methods dedicated to these aims. In conclusion, the inventory highlights promising but as yet underdeveloped areas of methodological development. For example, the number of organoid models identified is surprisingly limited. While we identified a larger number of microfluidic systems, their scope shows substantial room for improvement as well. For example, future developments should include the optimisation of microfluidic devices for the identification of NDD-related biomarkers in clinical settings and as tools to monitor disease progression. As new in vitro amyloid β diagnostic devices continue to mature, they have the potential to enable broader implementation of disease screening and earlier diagnosis of asymptomatic AD patients. In addition, improved high-throughput screening of drug candidate interactions could be achieved, for example, in combination with organoids.

## 6. Conclusions

Reliance on animal models is failing to identify a pathway to novel therapies in biomedical research; thus, there is an increased interest in using in vitro, ex vivo and in silico approaches. EURL ECVAM published the results of a project that provides an inventory and scientific evaluation of innovative (human-based) non-animal models/approaches currently in use for basic and applied research.

Stakeholder groups targeted include biomedical scientists interested to apply non-animal models in a particular disease area, National Committees (Ethics, Funding) and teaching institutions (universities). Promoting a platform useful for researchers, evaluators and policymakers could be the focus of concerted efforts to satisfy the Three Rs in biomedical research and, thus, move closer to achieving the ultimate aim of Directive 2010/63/EU.

## Data Availability

https://data.jrc.ec.europa.eu/dataset/176d71e6-5082-4b29-8472-b719f6bda323 (accessed on 19 July 2022), https://data.jrc.ec.europa.eu/dataset/ffebe454-ed9a-47cf-8a33-8cf70c1b7d38 (accessed on 19 July 2022).
